# Lessons from an ICU recovery clinic: two cases of meralgia paresthetica after prone positioning to treat COVID-19-associated ARDS and modification of unit practices

**DOI:** 10.1186/s13054-020-03289-4

**Published:** 2020-09-27

**Authors:** Amy L. Bellinghausen, Jamie N. LaBuzetta, Frank Chu, Francesca Novelli, Anthony R. Rodelo, Robert L. Owens

**Affiliations:** 1 Division of Pulmonary, Critical Care, Sleep Medicine and Physiology, University of California San Diego, San Diego, CA USA; 2 Department of Neurosciences, Division of Neurocritical Care, University of California San Diego, San Diego, CA USA; 3 Department of Pharmacy, University of California San Diego, San Diego, CA USA; 4 Critical Care Nursing, University of California San Diego, San Diego, CA USA

**Keywords:** Meralgia paresthetica, Prone, COVID-19, Acute respiratory distress syndrome, ICU recovery clinic

## Main text

Prone positioning is one of the few interventions in acute respiratory distress syndrome (ARDS) which has a proven mortality reduction [1]. Due to the coronavirus disease 2019 (COVID-19) pandemic, severe ARDS cases have sharply increased worldwide, increasing the need for proning. Some centers have also encouraged non-intubated patients with hypoxemia due to COVID-19 to self-prone [2]

**Fig. 1:**
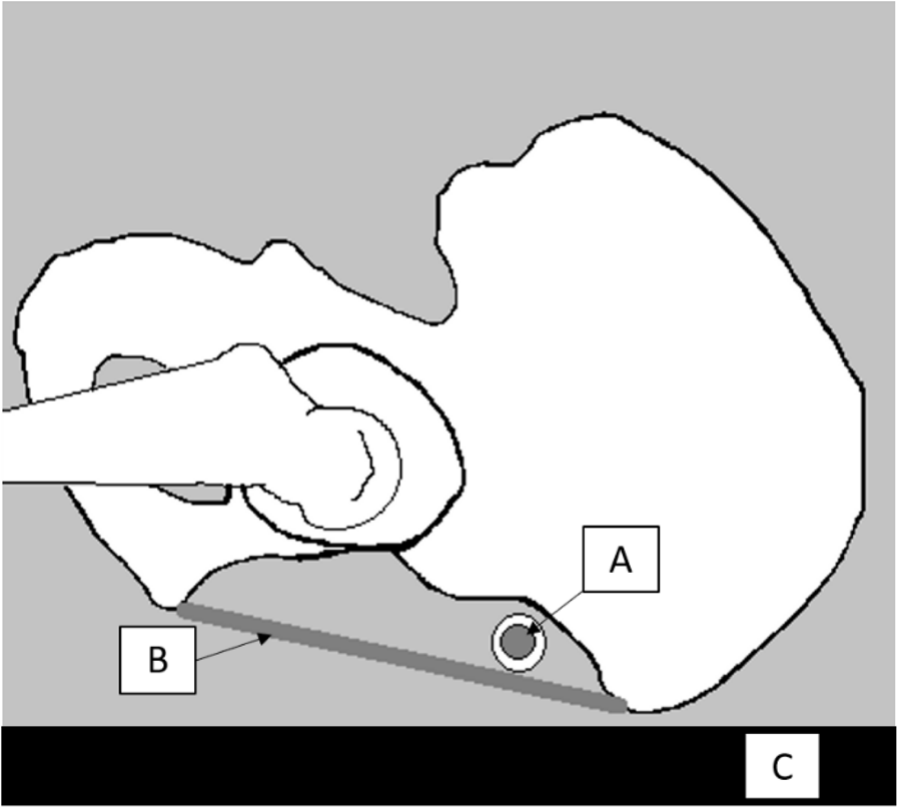
Lateral femoral cutaneous nerve compression in the prone position. (A) Lateral femoral cutaneous nerve (cross-section). (B) Inguinal ligament. (C) Surface of the hospital bed

Although generally considered low risk, pressure-related complications can occur during proning and differ from those that occur in supine patients. We present two cases of COVID-19-associated ARDS treated with prone positioning who developed meralgia paresthetica that was diagnosed in our ICU recovery clinic. Meralgia paresthetica (MP) results from compression injury of the lateral femoral cutaneous nerve between the anterior superior iliac spine and the inguinal ligament (Fig. 1); this mononeuropathy results in sensory abnormalities in the anterolateral thigh [3]. To our knowledge, there is only one other reported case of MP in prone positioning for ARDS, although it has been reported after surgical prone positioning in up to 24% of cases [4, 5].

The first patient was a 53-year-old man with diabetes (well-controlled, glycosylated hemoglobin 6.5 to 7.0%), obesity (BMI 30.9), and hypertension, who was mechanically ventilated for 11 days. He had a single session of proning for 16 h. He was extubated and discharged home on day 19 of admission. During his ICU recovery clinic visit (2 months after discharge), he reported isolated left-sided, well-demarcated anterior thigh numbness, new since his hospital stay. He had no associated weakness or pain, though did also endorse some non-painful numbness and tingling in the bilateral second and third toes, which was also new since discharge, without associated weakness, thought to be potentially due to peroneal nerve compression. As the symptoms were not troublesome to the patient, further electrophysiological testing was deferred.

The second patient was a 57-year-old man, without known past medical history who was intubated, mechanically ventilated, and proned for a total of 42 h, over three sessions. He was extubated after 10 days and discharged home on day 24 of admission. At his ICU recovery clinic visit (7 weeks after discharge), he endorsed left anterior thigh numbness without associated weakness, which was bothersome though not painful. His primary care provider had prescribed capsaicin cream (which depletes substance P, reducing nerve sensitivity to painful stimuli), which had not improved the symptoms. The patient declined referral for electrophysiological examination.

**Fig. 2:**
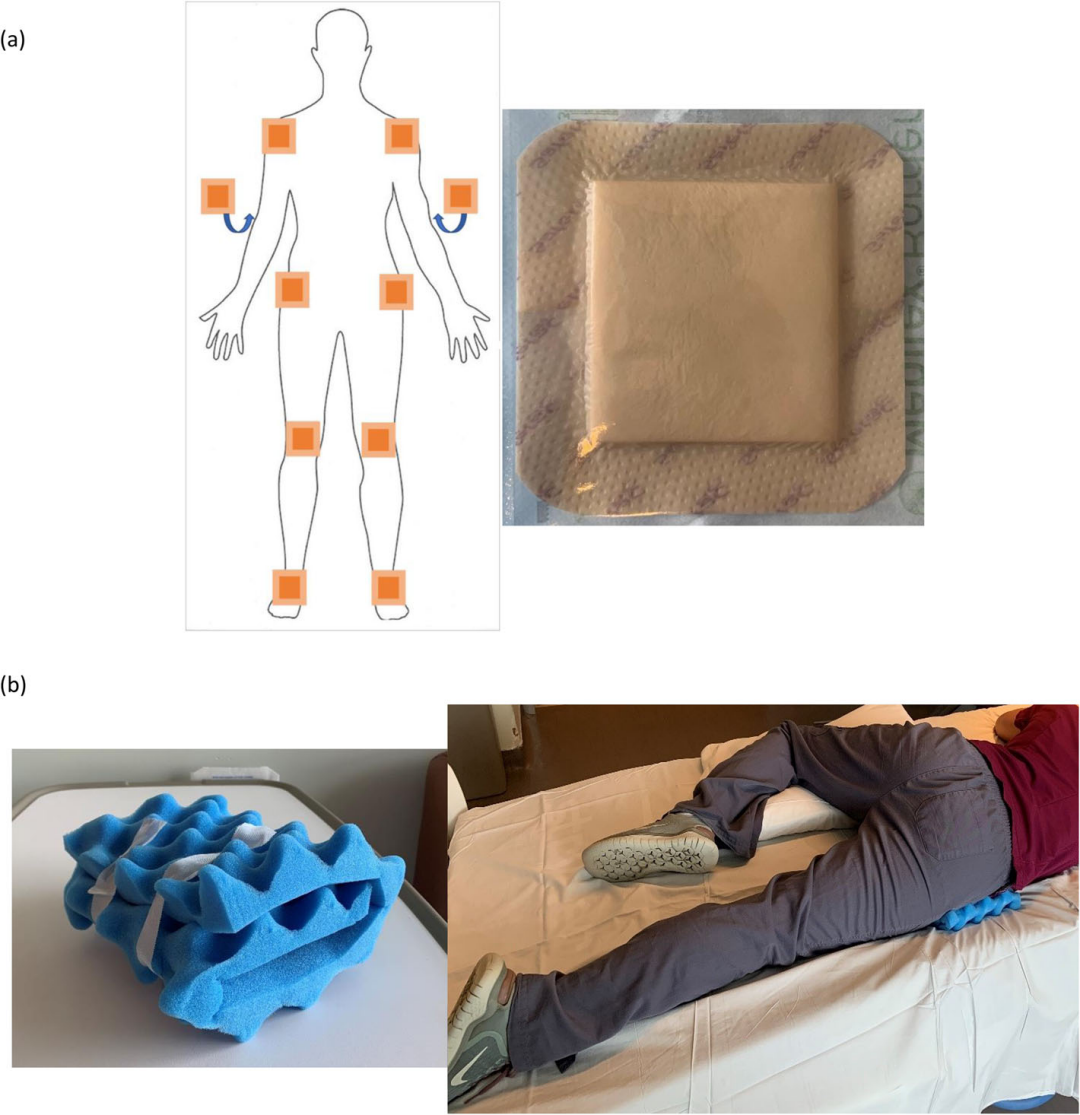
**a** Absorbent, adhesive, foam dressings (10 cm × 10 cm) and locations used for the initial pressure offloading technique. **b** Addition of egg-crate foam under the “down hip” in revised pressure offloading technique

After review of these cases, we altered our unit practice regarding padding of the anterior hips while patients are in the prone position. Previously, we placed 4-in. square, adhesive, foam absorbent dressings over each anterior superior iliac spine, and a pillow under the “up” hip (alternating sides every 2 h). Subsequently, we have changed our practice to more evenly distribute pressure over the hip, with egg-crate style foam padding between the “down” hip and the bed (Fig. 2).

We present these cases not only to highlight an uncommonly recognized but potentially preventable complication of prone positioning in the ICU, but to show how data gathered in an ICU recovery clinic, staffed by intensivists, can be “fed back” into the ICU where patients were treated, and improve the quality of care that patients receive [6]. “Identifying otherwise unseen targets for ICU quality improvement” has been postulated as one way that ICU recovery clinics might improve care, yet there are few published examples. If these patients returned to their primary care physicians, it is less likely that the cause of the MP would be known, nor would practice change. Lessons like these show the potential value of ICU recovery clinics, not only in treating post-intensive care syndrome, but in changing its underlying causes.

## Data Availability

Not applicable

## Abbreviations

COVID-19: Coronavirus disease 2019
ARDS: Acute respiratory distress syndrome
ICU: Intensive care unit
MP: Meralgia paresthetica
